# Contrasting drivers of abundant phage and prokaryotic communities revealed in diverse coastal ecosystems

**DOI:** 10.1038/s43705-023-00333-6

**Published:** 2023-12-04

**Authors:** Alaina R. Weinheimer, Frank O. Aylward, Matthieu Leray, Jarrod J. Scott

**Affiliations:** 1 Department of Biological Sciences, Virginia Tech, Blacksburg, VA USA; 2 Bigelow Laboratory for Ocean Sciences, East Boothbay, ME USA; 3 Center for Emerging, Zoonotic, and Arthropod-borne Pathogens, Virginia Polytechnic Institute and State University, Blacksburg, VA 24061-0913 USA; 4 Smithsonian Tropical Research Institute, Balboa, Ancon Republic of Panama

**Keywords:** Microbial ecology, Bacteriophages, Microbial biooceanography

## Abstract

Phages (viruses of bacteria and archaea) are a ubiquitous top-down control on microbial communities by selectively infecting and killing cells. As obligate parasites, phages are inherently linked to processes that impact their hosts’ distribution and physiology, but phages can also be impacted by external, environmental factors, such as UV radiation degrading their virions. To better understand these complex links of phages to their hosts and the environment, we leverage the unique ecological context of the Isthmus of Panama, which narrowly disconnects the productive Tropical Eastern Pacific (EP) and nutrient-poor Tropical Western Atlantic (WA) provinces. We could thus compare patterns of phage and prokaryotic communities at both global scales (between oceans) and local-scales (between habitats within an ocean). Although both phage and prokaryotic communities differed sharply between the oceans, phage community composition did not significantly differ between mangroves and reefs of the WA, while prokaryotic communities were distinct. These results suggest phages are more shaped by dispersal processes than local conditions regardless of spatial scale, while prokaryotes tend to be shaped by local conditions at smaller spatial scales. Collectively, we provide a framework for addressing the co-variability between phages and prokaryotes in marine systems and identifying factors that drive consistent versus disparate trends in community shifts, essential to informing models of biogeochemical cycles that include these interactions.

## Introduction

Phages, viruses that infect prokaryotes bacteria and archaea, modulate the ecology and evolution of microbial communities through selectively killing cells, horizontally transferring genes between cells, and reprogramming cell metabolism during infections [1]. Understanding the impacts of phages on prokaryotes is critical toward modeling the movement of nutrients through ecosystems [2], the evolution of prokaryotic pathogens [3], and the dynamics of organismal-associated microbiomes [4]. While rapid advances in sequencing and microscopy technologies over the past few decades have begun to unfold the vast diversity, complexity, and breadth of viruses in nature [5–7], major questions remain on which factors shape phage communities and how this relates to concomitant shifts in prokaryotic communities.

Although viruses are limited to reproducing through their hosts, their compositional and diversity patterns may differ from their hosts’. For example, a study on the meeting point of a freshwater river and spring in Florida found that prokaryotic communities were distinct between different sampling points within 1 kilometer of each other, but phage communities were not distinct between the sampling points [8]. In soils, a study found that biochar treatment (4.5–55.4% ash content) shaped phage communities but not prokaryotic communities [9]. Several possibilities have been suggested to explain these contrasts. The importance of dispersal versus species local adaptation may differ between prokaryotes and phages [8]. Additionally, phages that have broader host ranges may be less impacted by changes in host composition [10]. More physically, virion particles can be degraded by UV light exposure [11], and extremes in pH (e.g. outside 5.7–7) can limit the ability of phages to attach to hosts [12], which could also decouple the relationship between phage and prokaryotic community patterns. Taken together, these studies highlight the need to examine factors shaping both phage and the corresponding prokaryotic communities to better untangle the impact of the environment on their interactions.

In this study, we leverage the unique biogeography of the Isthmus of Panama to uncover factors shaping viral and microbial communities across a diverse array of tropical, coastal environments in two oceans. The Isthmus of Panama gradually formed and completely disconnected the Tropical Western Atlantic Ocean (WA) from the Tropical Eastern Pacific Ocean (EP) approximately 2.8 million years ago [13]. The WA became oligotrophic, leading to the proliferation of reef-building corals. The EP remained eutrophic, with patchy coral reefs dominated by fewer species of scleractinian corals. Expansive mangroves thrive adjacent to coral reefs in both the EP and the WA. Nonetheless, mangroves of the WA are influenced by much smaller tidal oscillations than in the EP. In addition, the WA supports thinner fringes of mangroves made of shorter trees than in the productive EP [14]. These contrasting coasts with similar habitat types of mangroves and coral reefs allow comparisons of phage and prokaryotic communities at two spatial scales, locally among the habitat types within an ocean and globally between the oceans [15].

We focused on phages detected in metagenomes of cellular size fractions of seawater (>0.22 µm), as they represent the putatively abundant or active members of the phage community as indicated in previous studies [16, 17]. Given the intrinsic link of phages to their hosts, our null hypothesis was that factors shaping the communities of these abundant phages would mirror those shaping prokaryotic communities, and this similarity would be most visible at global scales between the oceans since the spatial separation and chemical differences between oceans are so large. An alternative hypothesis is that factors shaping phage communities would not match those of the corresponding prokaryotic communities, and these differences would be most apparent at smaller scales where subtle differences in environmental parameters can influence contact rates of phages to prokaryotes, growth rates of prokaryotes, and other physical aspects that may decouple phage communities from prokaryotic communities. Overall, our results highlight the impact of spatial scales on the strength of ecological relationships between phage and prokaryotic communities. Understanding the factors that impact whether phage and prokaryotic communities couple each other is crucial for modeling phage-host interactions as they relate to microbial mortality, and ultimately biogeochemical cycling in ecosystems.

## Results

### Benchmarking methods to assess phage and prokaryotic diversity and sampling site features

To directly compare phage and prokaryotic diversity and minimize information loss from the metagenomic data, we benchmarked and employed a gene-based approach (see Methods for details). Briefly, we focused on those of the Caudoviricetes class, as they corresponded to 99.7% of the phage sequences in our data (Supplementary Dataset 2). We examined major capsid protein (MCP) and terminase large subunit (TerL) sequences belonging to the Caudovircetes in all contigs. Additionally, we detected and compared their results with whole contigs detected as phages, to enable comparison with traditional virome approaches. Because ecological statistics held for all three phage sequence types (Supplementary Dataset 4), we report the results from the TerL here, as this was the most prevalent phage gene (Supplementary Dataset 4) and enabled direct comparison with prokaryotic single-genes (versus metagenome assembled genomes). Prokaryotic diversity was examined with protein sequences from families of three genes: RNA polymerase *β*, RNA polymerase *β*′, and a ribosome-binding ATPase YchF (COG12), as used in previous studies of prokaryotic communities [18, 19]. Ecological statistics held for all three genes (Supplementary Dataset 4), and the results of RNA polymerase β are reported here as this was the most prevalent gene in the dataset (Supplementary Dataset 4). Details on sequence detection can be found in the Methods to use this approach for other datasets and studies.

**Fig. 1:**
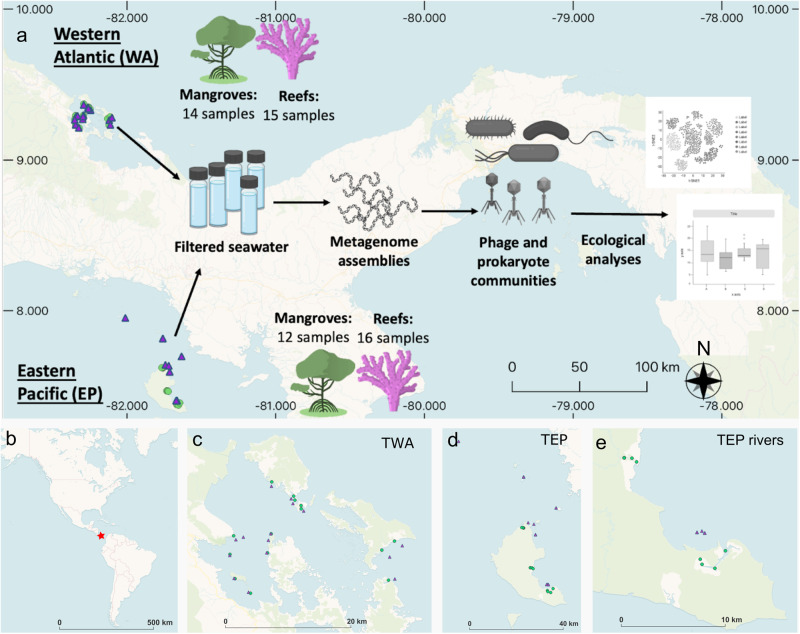
Overview of project design with maps of sample locations. **a** Graphical abstract of project approaches. **b** World map with Panama denoted as red star. **c** Map of sample sites from the Tropical Western Atlantic (WA) coast of Panama. **d** Map of sample sites from Tropical Eastern Pacific (EP) coast of Panama. **e** Map of EP mangrove samples zoomed in on those collected along two freshwater rivers and the nearby reef samples. Green circles are mangrove samples. Purple triangles are reef samples.

In total, fifty-seven samples of seawater from mangroves and reefs were collected from the WA and EP coasts of Panama and filtered on 0.22 µm pore filters that were processed for metagenomic sequencing (Fig. 1). Although most known phages are smaller than 0.22 µm, viral sequences comprised a substantial portion of the classified reads in these samples (Supplementary Dataset 1; average of 23.7%, 3.57% average of total reads), and phages in cellular size fractions of other studies have been shown to correspond to an abundant and putatively active subset of the total phage community [16, 17]. We thus referred to the phages of this study as belonging to the abundant subset of the total phage community.

### Variability between oceans was lower in prokaryotic communities compared to phage communities

**Fig. 2:**
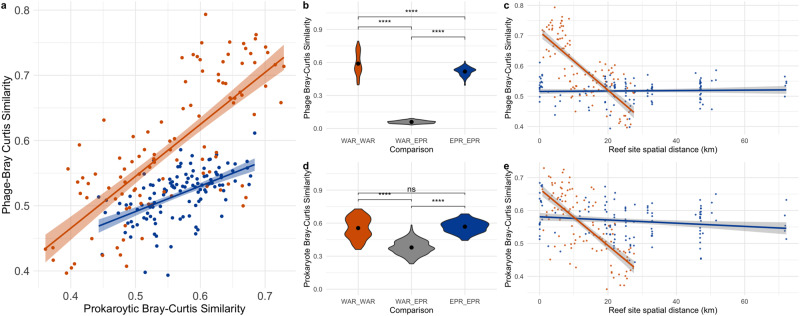
Comparisons of variation in composition of phage and prokaryotic communities between reefs of the Western Atlantic Reefs (WAR) and Eastern Pacific Reefs (EPR). **a** Phage Bray–Curtis similarity between samples within the WAR or EPR plotted against the prokaryotic Bray–Curtis similarity of those two samples. Points and lines are colored by ocean. Violin plots of phage (**b**) and prokaryotic (**d**) Bray–Curtis pairwise similarity of samples within the same ocean (EP_EP, WAR_WAR) or between oceans (WAR_EPR). Bray–Curtis similarity of the phage (**c**) or prokaryotic community (**e**) of two samples plotted against the distance between reef sites within the EPR or WAR. Colors correspond to ocean or ocean comparison of samples (WAR - orange, EPR - blue, Both oceans - gray). Lines correspond to linear regression with standard error shaded. Significance in boxplots indicated by stars with the following *p* values: **** < 0.0001, ns not significant.

To examine differences in communities between the oceans, we focused on the reef samples of the EP and WA samples since the mangrove samples in the EP were collected in freshwater rivers, which obfuscates direct comparison with the marine WA mangroves (Fig. 1). These reef samples are referred to as the EPR (Eastern Pacific Reef) or WAR (Western Atlantic Reef). Both phage and prokaryotic community compositions differed significantly between the two oceans (Bray–Curtis distances, PERMANOVA phage *p* values < 0.01; Supplementary Dataset 4). Within each ocean, the variation in community compositions of prokaryotes and phages correlated with each other (Fig. 2a; Mantel test of Bray–Curtis distance matrices *p values* < 0.01; Pearson correlation of Bray–Curtis distance *p* values < 0.01; Supplementary Dataset 4). Differences in phage composition between the EPR and WAR, however, differed to a larger extent than the prokaryotic composition differed. Half as many phages could be found in both oceans compared to the prokaryotes (12% versus 25%), and 64% of the variation in the reef phage communities could be attributed to ocean, compared to only 35.5% for the prokaryotic communities (PERMANOVA *R*^2^, Supplementary Dataset 4). Additionally, phage communities had an average similarity of 5.9% (Bray–Curtis Distance) between samples of different oceans, while prokaryotic communities had an average similarity of 37.9% between reefs of the different oceans (Fig. 2b, d). The high similarity of prokaryotic communities across oceans is in line with a recent study of beta diversity patterns in prokaryotic communities across the global ocean which found an average similarity of 38.9% across surface water samples [20]. The phages of this study were detected in the cellular fraction of seawater, which potentially corresponds to a more ephemeral but abundant subset of the total phage community, as has been found in a study on cyanophages in a coastal bay [17] and may explain their limited distribution compared to the prokaryotes.

To further determine the dispersal of these reef phages and prokaryotes across the global ocean, we examined the presence of these prokaryotes and phages in samples from the global ocean sampling effort Tara Oceans [21] (see “Methods”; Supplementary Dataset 3; Supplementary Fig. 1). Specifically, we examined Tara Oceans metagenomes of the cellular size fraction (0.22-3 μm), as these samples most closely match our study’s filtering. Collectively, the prokaryotes were found in significantly more Tara Ocean samples than were the phages (Wilcox test *p* value < 0.01; Supplementary Fig. 2; Supplementary Dataset 5). The average number of samples that a prokaryote was detected in was 4.25, while the average number of samples for phages was 0.94. Two of the Tara Oceans samples were collected near Panama’s coasts, with samples from Tara station 141 near the Atlantic coast and those from Tara station 140 near the Pacific coast. A little more than one-fourth of prokaryotes were found in both of these stations (27.5% and 26.2%, respectively), and 27.7% of prokaryotes that were detected in both the WAR and EPR were found at these stations. Meanwhile, much fewer phages could be found at these stations (12% and 12.9%, respectively), and only 12.5% of phages that were detected in the EPR and WAR were found at these stations. Again, the limited detection of phages at these Tara stations relative to prokaryotes is likely because the cellular fraction was examined to be comparable with our samples, which both exclude less abundant phages [9]. The limited detection of phages across oceans, however, was also observed in a global ocean survey of viral diversity in size fractions below <0.22 μm, in which phages would be enriched [22]. This study that examined viruses of viral size fractions found that viruses of tropical and temperate latitudes (like those of this study) were mostly endemic to the oceanic region of the metagenomic in which they were initially recovered (e.g. North Atlantic Ocean or South Pacific Ocean). Additionally, only ~20% of these viruses could be found in other regions. Thus, both viruses of viral size fractions and viruses of cellular size fractions (shown in this study) have limited global distribution, suggesting the overall dispersal of viruses may be more restricted than that of prokaryotes.

To examine dispersal at the community-level, we compared how much the community similarity changed as the distance between reef sites within each ocean increased using a distance decay analysis (Fig. 2c, e). Within the WAR, the similarity of communities between sites significantly decreased with increasing distances between sites for both phages and prokaryotes (Fig. 2c, e; Pearson correlation of Bray–Curtis similarity against spatial distance, *p* valu*e* < 0.01). Meanwhile, in the EPR, community similarity did not significantly change as distance between sites increased (Pearson correlation of Bray–Curtis dissimilarity and spatial distance, *p value* < 0.01) for neither phages nor prokaryotes (Fig. 2c, e). This lack of distance decay in community similarity of the EPR is surprising because the distance between sites and environmental conditions (pH, salinity, temperature, dissolved oxygen) were much more disparate in the EPR compared to the WAR (Supplementary Dataset 1). For instance, the EPR sites spanned up to 60 kilometers while the WA reef sites spanned less than 30 kilometers. As mentioned, the EP coast experiences stronger tidal oscillations, or mixing, than the WA coast [15], and thus, the lack of shift in phage and prokaryotic communities in the EP across larger environmental and spatial distances suggests that the strength of this mixing is more important than differences in local conditions in shaping phage and prokaryotic communities here. The disparate impact of geographic distance on communities in the WAR versus EPR highlights the variability in contribution of distance and local conditions on structuring phage and prokaryotic communities, as has been observed for phages and prokaryotes in other aquatic systems [8, 20, 23].

### Distinct drivers of alpha diversity in prokaryotic and phage communities

**Fig. 3:**
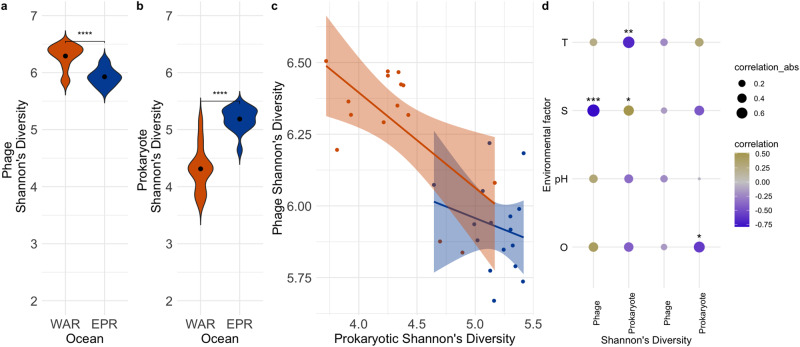
Patterns of community diversity in the WAR and EPR for phages and prokaryotes. **a**, **b** Shannon’s Diversity between both oceans. **c** Phage versus prokaryotic Shannon’s Diversity in the EPR (blue) and WAR (orange) with a linear regression line and standard error shaded. **d** Correlogram of phage and prokaryotic diversity against different environmental parameters in the WAR (left) and EPR (right). T temperature, S salinity, O dissolved oxygen.

Although the phage and prokaryote communities generally aligned with each other in compositional shifts between the EPR and WAR, patterns in their community diversity contrasted each other. Phages were more endemic to the WAR than the EPR (49.7% vs. 37.7%) and significantly more diverse in the WAR (Fig. 3a) (Shannon’s Diversity Wilcox test *p* values < 0.01). Meanwhile, prokaryotes were more endemic to the EPR than the WAR (50.9% vs. 25.4%) and significantly more diverse in the EPR than the WAR (Fig. 3b) (Shannon’s Diversity Index Wilcox Test *p* value < 0.01). In fact, phage diversity did not significantly correlate with prokaryotic diversity in neither the EPR nor WAR (Fig. 3c; Pearson correlation of Shannon’s Diversity Indices *p* values > 0.01; Supplementary Dataset 4). Consistent with this, a previous study examined phage and prokaryotic diversity in the mesopelagic ocean (below 200 meters) and found that their diversities did not significantly correlate [22], although they did find possible links when examining fine-scale patterns of phage microdiversity (i.e. nucleotide variation within populations containing >95% ANI).

Nevertheless, several studies have observed that similar features increasing phage diversity also increase prokaryotic diversity in marine environments, such as temperature [22] and depth [19]. We thus examined whether available environmental data (salinity, temperature, dissolved oxygen, and pH) correlated similarly with phage and prokaryotic diversity, despite the observed lack of correlation with each other (Fig. 3d). In the WAR, phage diversity correlated with each parameter in the opposite direction that prokaryotic diversity did. Among the significant correlations (Pearson correlation *p* value < 0.05), phage diversity negatively correlated with salinity, while prokaryotic diversity positively correlated with salinity. Similarly, prokaryotic diversity negatively correlated with temperature significantly (*p* value < 0.05), while phage diversity positively correlated with temperature albeit not significantly (*p* value > 0.05). Meanwhile, in the EPR, phage diversity appeared to negatively correlate with all variables though not significantly, and prokaryotic diversity negatively correlated with all variables except temperature, but this was not significant. The apparent opposition of drivers of phage and prokaryotic diversity in the WAR versus their general concordance in the EPR is striking considering the magnitude of environmental differences were higher in the EPR than the WAR (Supplementary Dataset 4). For instance, salinity ranged more in the EPR (2.44 ppt) than in the WAR (1.66 ppt) as did dissolved oxygen (2.24 mg/L versus 1.97 mg/L). Perhaps other environmental variables not measured in this study (e.g. nutrients, turbidity) may be impacting phage and prokaryotic diversity in the EP, leading to these insignificant correlations. Nonetheless, taken together, these results show that factors driving abundant phage and prokaryote diversity rarely align even though variation in their composition may correlate.

### Variation in communities between benthic habitat types was greater for prokaryotes than phages

**Fig. 4:**
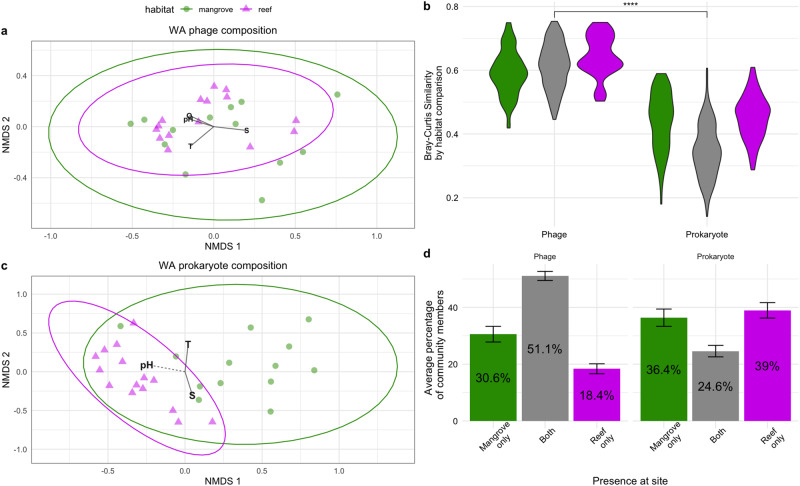
Variation in phage and prokaryote communities between the WAR and WAM. NMDS plot of phage (**a**) and prokaryotic community compositions (**c**) based on Bray–Curtis dissimilarities in the WA colored by habitat type. Ellipses are drawn based on a normal multivariate distribution. Significant environmental factors that correlated with compositional variation are shown with solid lines corresponding to *p* values < 0.01 and dashed lines for *p* values < 0.05. **b** Violin plot of Bray–Curtis similarity between samples within the WAM (green) between the WAR and WAM (gray) and within the WAR (pink). Significance bar stars correspond to a *p* values < 0.001 from Wilcox tests of average similarities of phage and prokaryotic communities. **d** Barcharts of the percentage of community members found at a site that was either present in only the mangrove of the site (green), the reef of the site (pink) or both (gray). Error bars correspond to standard error.

To determine how benthic habitat type can impact phage and prokaryotic communities of the associated water column, we focused on samples collected from mangroves and reefs within the WA. The mangrove samples are referred to as belonging to the WAM. Phage communities varied less between the habitat types than did the prokaryotic communities (Fig. 4a, c). When comparing their composition across habitat types, phage communities did not significantly differ (PERMANOVA *p* value > 0.01) and only 6.4% of variation attributed to habitat type, while the prokaryote community compositions significantly differed with 21.8% of their variation explained by habitat type (PERMANOVA *p* value < 0.01). Furthermore, the phage communities had significantly higher similarity when comparing the Bray–Curtis similarity of samples between the two habitat types (Fig. 4b; phage mean 61.4%, prokaryote mean 34.8%; Wilcox test *p value* < 0.001). The lack of distinction of phage communities between habitat types suggests a higher dispersal of phages across habitat types than prokaryotes, which has been similarly observed in a lotic system where prokaryotic communities were distinguished between the different points in the spring system, but the phage communities were indistinguishable [8].

To investigate this divergence in distinction between the WAM and WAR further, we examined the presence of phages and prokaryotes across the reef and mangrove samples belonging to the same site. Twelve of the WAM and twelve of the WAR samples were collected in pairs at the same site (Fig. 1b). WAM and WAR samples of the same site had a maximum distance of 1.6 km between each other (Supplementary Dataset 1). When examining the presence of phages and prokaryotes across the habitat types at a single site, on average, over half of the phages (51.8%) were present in both the mangrove and reef of a site (Fig. 4d), while roughly one-fourth of prokaryotes (24.6%) were detected in both the mangrove and reef of a site on average (Fig. 4d). This high dispersal of phages across habitats within a site contrasts the freshwater springs study, which found that even though phage communities were indistinguishable between sites, phages were more endemic to a single site than prokaryotes were [8]. Nevertheless, although phages appear less sensitive to habitat type than prokaryotes given the above analyses, variation in the phage and prokaryotic communities significantly correlated with each other (Mantel test of Bray–Curtis distance matrices *p* value < 0.05; Supplementary Dataset 4), suggesting shifts in the available host community still impact phage community composition even though distinctions between habitat types are not apparent.

When examining patterns of community diversity, there was no clear evidence that benthic habitat impacted phage or prokaryotic Shannon’s Diversity, as they were both equally diverse across the WAR and WAM (Wilcox test *p* values > 0.05, Supplementary Dataset 4). Furthermore, phage and prokaryotic diversity correlated with environmental factors in the same way they had in the WAR samples (Fig. 3d; Supplementary Fig. 3). Taken together, benthic habitat type in the WA structures prokaryotic community composition moreso than it structures phage composition but does not seem to impact patterns of diversity for either of these communities. The lack of alpha-diversity differences for prokaryotes despite differences in composition is not unique to the WAR, as it has been observed in a global study of bacterial communities that compared saline and non-saline habitats in which composition differed with salinity but not alpha diversity [24]. In these cases, the environments being compared may be selecting for different organisms resulting in compositional differences, but they are able to support similar levels of diversity within their communities, resulting in similar alpha-diversity levels.

### Phage and prokaryotic compositions differed similarly between the EP mangrove rivers

**Fig. 5:**
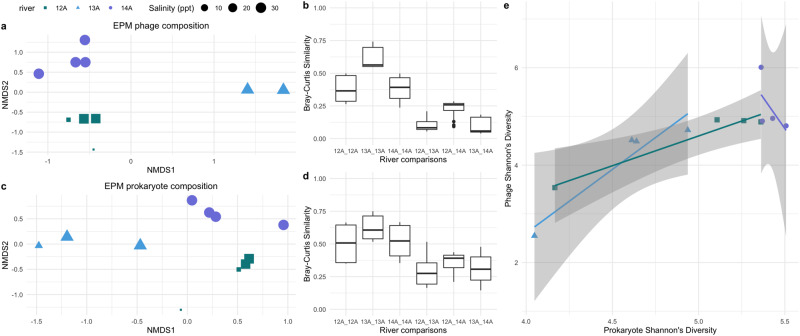
Compositional and diversity patterns of phage and prokaryote communities in the EPM. NMDS plots of the phage (**a**) and prokaryote (**c**) community composition based on Bray–Curtis distances. Points are colored by river and size corresponds to salinity in ppt. Some points overlapped (e.g. 13A2 and 13A3 in **a**). Boxplots of Bray–Curtis similarity between samples belonging to the same or different rivers for phage **(b**) and prokaryotic (**d**) communities. Rivers compared are separated with an underscore. **e** Shannon’s Diversity of phages in a sample plotted against that of prokaryotes in the EPM color and shape by river. Line calculated from linear regression. Standard error is shaded.

The EP mangrove samples (EPM) were collected at four points along three rivers, of which two were along a fresh water gradient (samples 12A1-4 and 13A1-4), and one river was completely saline (samples 14 A1-4) (Supplementary Dataset 1). Due to the unique spatial and salinity features of these samples compared to the other environments of this study (EPR, WAR, WAM), we examined the ecology of these samples only in relation to each other. Among the EPM samples, the phage and prokaryotic community composition of freshwater sample 13A1 was particularly aberrant and excluded from compositional analyses (Supplementary Fig. 4). When examining the communities of the remaining samples, the phage and prokaryotic community compositions appeared to cluster by river rather than by salinity (Fig. 5a, c), and both phage and prokaryotic communities of the same river had higher similarity than with those of different rivers (Fig. 5b, d). Despite salinity being a key driver of microbial communities [25, 26], surprisingly, the communities within the fully marine 14A river had similar variation between each other as river 12A, which spanned in salinity from 0.06, 2.74, 26.37, to 28.01 ppt, further highlighting the importance of river separation over salinity in structuring these communities.

Both phage and prokaryotic communities significantly varied between rivers to a similar magnitude, in which 65.6% and 60.7% of the variation in the communities were explained by river for the phage and prokaryotes, respectively (PERMANOVA *p* values < 0.01; Supplementary Dataset 4), and variation in their compositions correlated with each other (Mantel test of Bray–Curtis distance matrices *p* value < 0.01; Supplementary Dataset 4). Although few riverine virus-prokaryote studies exist to date [8, 27, 28], alignment in compositional variation of DNA viruses and bacteria has been observed in freshwater rivers of southwestern British Columbia [28].

Regarding community diversity, phage and prokaryotic Shannon’s diversity also significantly correlated positively with each other in the rivers with the freshwater gradient (12A,13A), but not in the saline river (14A) (Fig. 5e), and they both positively increased with salinity in rivers 12A and 13A, though few sample points were available for statistical testing. Overall, the saline river 14A varied less in salinity and temperature compared to rivers 12A and 13A (Supplementary Dataset 1), which are known drivers of phage and prokaryotic communities [21, 22] and may explain why little correlation in their diversities could be seen in the river 14A. That being said, there are very few sampling points to generalize this finding, especially as drivers of microbial communities have been found to vary substantially between rivers [28] (Fig. 5b, d; Supplementary Dataset 4). Although limited in sampling points, these findings of the EPM rivers contribute to the overall paucity of riverine phage-prokaryote studies.

#### The most prevalent and influential phages and prokaryotes distinguishing the communities belong to diverse taxa and ecological groups

To determine which groups of phages and prokaryotes were driving the distinctions in the composition of communities, we classified the sequences using multiple approaches. The phages were classified based on the taxonomy of their putative host estimated by the alignment of the terminase large subunit (TerL) sequences to genes of RefSeq 207 [29] and examining the host of the hits. RNA polymerase beta subunit (RNAP β) sequences used to represent prokaryotic diversity here were classified based on the consensus classification of the contig on which the RNAP β was present (Supplementary Dataset 5; See Methods for details).

Of the top ten most prevalent genera based on average relative abundance across samples, only three genera overlapped for prokaryotes and putative phage hosts: *Synechococcus*, *Prochlorococcus*, and *Pelagibacter* (Supplementary Fig. 5; Detailed discussion in Supplementary Information). These genera are known as dominant members of the ocean [30, 31]; furthermore, because the phage sequences may also correspond to integrated phages of the prokaryotic community, this may have resulted in the co-prevalence of these genera in both phage and prokaryotic communities. Nevertheless, the general lack of overlap in prevalent phage and prokaryotic genera may have resulted from several factors such as technical limitations in classifying both the phages and prokaryotic sequences or that most viral lysis occurs for rare but highly productive microbes, as has been observed off the coast of British Columbia in Canada [32], which would result in dominant viruses that infect rarer hosts.

We then examined which phages and prokaryotes drove the most variation between the samples, which was determined by those that significantly varied the most with variation in the communities of the WA and EP separately (envfit test; *p values* < 0.05; See “Methods”; Supplementary Dataset 5). In the WA, the two phages that drove most of the variation showed high homology to the terminase of *Pelagibacter* phage HTVC008M and the *Puniceispirillum* phage HMO-2011, prokaryotic genera that are both heterotrophic bacteria found throughout the global ocean [30, 33]. The prokaryotes driving the most variation in the WA communities belonged to genera of an uncultivated genus WTJO01 in the Puniceispirillales order, and the next most influential belonging to an uncultivated genus UBA974 in the Flavobacteriales order. These heterotrophic bacteria are also found throughout the oceans [30, 34]. The alignment in taxa of the prokaryote and putative host of phages driving differences between the communities of the WA is surprising because their overall taxonomic composition did not align (Supplementary Fig. 5).

Within the EP, the most influential phages primarily putatively infect bacteria belonging to the photosynthetic *Synechococcus* genus (seven of the top ten), while the most influential prokaryotes primarily belonged to unknown genera in the Betaproteobacteria class (Supplementary Dataset 5). Although these genera contrast each other in trophic lifestyles, these bacteria are known to be highly influenced by salinity [35, 36], which widely varied among the EP as the mangrove samples that were collected along freshwater rivers. These results suggest that while phage and prokaryotic communities both vary substantially with salinity, the types of bacteria and putative hosts of phages that are most affected by salinity in these sites do not necessarily align.

## Conclusions

This study provides a framework for comparing phage and prokaryotic community composition and diversity in a variety of marine environments. Our analysis focused on the phages of the Caudoviricetes class as representative of phage patterns due to the very few representatives of other types of phages in the data. These Caudoviricetes phages were detected in a cellular fraction of seawater (>0.22 µm), which is known to exclude some rare but persistent viruses of the environment [9, 17] but nevertheless corresponds to the abundant and potentially active or lysogenic subset of the phage community which may be responsible for the infective interactions that drive prokaryotic communities [16]. Considering these phages are potentially more directly interacting with the prokaryotic community than those in the viral fraction, we expected that phage community patterns would closely align with that of prokaryotes. While this was generally the case between the EP and WA reefs and between the rivers of the EP mangroves, phage communities tended to be less distinct between the mangroves and reefs of the WA. Most phages could be found in both the mangrove and reef of a site, while few prokaryotes were found in both, suggesting that phages may be impacted by dispersal more than prokaryotes are when spatial scales are small such as between these WA sites. Similarly, a recent study on microbial communities in the global ocean found that prokaryotic communities were shaped more by local conditions compared to pico-eukaryotic communities which were more shaped by dispersal [23]. The relative importance of dispersal for structuring both pico-eukaryotic and phage communities compared to prokaryotic communities is an intriguing similarity given their distinct biologies and modes of replication. The underlying reasons for the importance of dispersal over local conditions likely differ but may have important implications in the ability of these communities to adapt to sea warming and acidification [37, 38].

In summary, this study highlights the importance of environmental factors in determining the relationship between the community composition and diversity of prokaryotic and abundant phage communities. We found that spatial separation, such as between oceans or rivers, tends to result in similar compositional patterns, but between more adjacent environments, such as the mangroves and reefs of this study, prokaryotic communities tend to be more structured by local conditions and phage communities appear to more structured by dispersal. By understanding when the links of phage and prokaryotic communities are strengthened or weakened, we can better predict the outcome of interactions between phages and prokaryote populations of different environments to inform models of nutrient cycling mediated by microbes and the release of organic matter through viral lysis of microbes.

## Methods

### Sample and environmental data collection

Seawater samples were collected ~1 m above the seafloor on coral reefs and mangroves (1–4 m depth) in the EP and WA coasts of Panama in 2017 (see Supplementary Dataset 1 for coordinates and collection dates). Dissolved oxygen, temperature, salinity, and pH were measured with a pre-calibrated Professional Plus handheld YSI (Yellow Springs, USA). Seawater samples for sequencing were collected in sterile Whirl-Pak Bags and kept on ice and in the dark until filtration at either the Smithsonian Tropical Research Institute (STRI) Coiba (EP) or Bocas del Toro research stations (WA), where they were then vacuum filtered through 0.22 µm nitrocellulose membranes with a 47 mm diameter (Millipore). Four liters of seawater were filtered for each sample, except for WAM_TWN, in which only 1 liter was filtered because it clogged the filter. Filters were frozen and transported to STRI’s molecular facility at Isla Naos Laboratory in Panama City in liquid nitrogen and stored at −80 °C until DNA extractions. Sampling to storage took no longer than four hours. DNA was extracted from each filter using a Qiagen Powersoil extraction kit following the manufacturer’s protocol with minor modifications to increase the yield [39]. Metagenomic shotgun libraries were prepared with the Illumina DNA Nextera Flex kit following the manufacturer’s protocol. Shotgun metagenomics reads were sequenced on an Illumina Nextseq 2000 platform at a depth of 10 million reads per sample.

### Metagenome preparation, sequencing, and assembly

We used Trimmomatic (v0.39) [40] for adapter clipping and initial quality trimming of raw metagenomic data (*N*  =  57). We used anvi’o (v7.1) [41] to build a Snakemake (v.5.10.0) [42] workflow for co-assembly analysis. In the workflow, we used iu_filter_quality_minoche from the Illumina Utils package (v2.12) [43] for additional quality filtering and MEGAHIT (v1.2.9) [44] for co-assembly (–min-contig-len: 1000, –presets: meta-sensitive). We performed three separate co-assemblies using MEGAHIT of the metagenomic data. The first co-assembly included all WA samples (reef and mangrove) (*n* = 29). In the EP, due to the wide range of salinity among the mangrove samples, we performed one co-assembly for mangrove samples (*n* = 12) and another for the reef samples (*n* = 16). Next, we used anvi-gen-contigs-database to generate a database of contigs. Within the Snakemake workflow, KrakenUniq (v0.5.8) [45] was used for taxonomic classification of short reads against a user-constructed database of archaea, bacteria, viral, fungi, and protozoa reads from RefSeq [29] and the NCBI nt database. Taxonomic classification of contigs was performed using Centrifuge (v1.0.4_beta) [46], against the bacterial, archaeal, human, and viral genomes database.

### Phage marker gene and contig curation

For the marker gene detection, open reading frames (ORFs) were predicted with prodigal [47] (-p meta -a -d) on contigs of all sizes (753,612 EP; 574,304 WA contigs | 2,168,906 EP; 1,756,476 WA ORFs; 3,925,382 total ORFs). Amino acid sequences of the ORFs were then searched against all MCP and TerL Hidden Markov Model (HMM) profiles available in Virus Orthologous Group database built on reference sequences belonging to the Caudoviricetes (vogdb.org) version 208 (Supplementary Dataset 2) using hmmsearch (hmmer.org; *E* value < 0.00001, bitscores >41 and >33, respectively, minimum length of open reading frame **≥**826 and **≥**885 nucleotides, respectively). The threshold bitscores were determined by searching proteins predicted with prodigal (default per genome) from all *Caudovirales* genomes from Viral Genomes Portal downloaded on July 26, 2021 against the MCP and TerL profiles, taking the top hit from each genome and identifying the minimum bitscore required to include at least 98% of hits. After filtering for bitscore, the minimum length of a hit was decided based on containing at least 98% of those reference hits. This resulted in 3749 MCP genes and 5369 TerL genes. These were then de-replicated at 100% nucleotide identity across the entire length of one sequence using BLASTn [48], which resulted in 3722 representative MCP and 5350 TerL (See “Data availability”).

For the detection of phage contigs, contigs over 10 kilobases (7619 EP; 10,839 WA) were run through VirSorter2 [49] and CheckV [50] as follows. First, contigs over 10 kilobases (EP: 7619, WA: 10,839;) were run through VirSorter2 (virsorter run --min-score 0.5 all) and retained if they scored over 0.5 for dsDNAphage as their max_group (EP: 1513, WA: 3272). These contigs were then run through CheckV (checkv end_to_end) to trim potential host genomes flanking the contigs. Trimmed provirus and virus sequences were combined and filtered for at least 10 kb (EP: 1482, WA: 3203). The trimmed sequences were then run through VirSorter again and retained if they scored over 0.95 or scored at least 0.5 and encoded at least 2 phage hallmark genes. This resulted in 3885 contigs. Virus detection summary for each contig is in Supplementary Dataset 2.

### Prokaryote marker gene curation

The same ORF and amino acid sequences used for the phage marker gene detection were searched against HMM profiles corresponding to genes to the Clusters of Orthologous Groups (COG) protein families of COG0012 (COG12, ribosome-binding ATP-ase), COG0085 (COG85, RNA polymerase β subunit), and COG0086 (COG86, RNA polymerase β′ subunit) [51] jointly using hmmsearch (*E* value < 0.00001, bitscores cutoffs of 210, 200, and 200, respectively [52]. See Supplementary Dataset 4 for the number of amino acid sequences aligned to each marker gene’s HMM profile.

### Distribution detection

Reads from all samples were subset to an even depth to the number of reads in the sample with the fewest reads (2,992,107 reads) with SeqKit [53] sample (-s 1000, -2). Reads were then mapped to an index of the phage marker genes, phage contigs, and prokaryote marker genes made with Minimap2 [54] (options: -x sr). CoverM [55] was then used for the mapping (coverm contig --min-read-percent-identity 95 -m covered_fraction rpkm count variance length --minimap2-reference-is-index --min-covered-fraction 0 --coupled) and retained with 50% gene covered or 20% of contig covered [56] (Supplementary Dataset 3). See Supplementary Dataset 4 for the number of each sequence type detected in at least one sample.

To examine the distribution of these phages and prokaryotes across the global ocean, we mapped reads from metagenomes of the 0.22–3 µm size fraction of seawater samples collected by Tara Oceans published in Sunagawa et al. [21] (sample information in Supplementary Dataset 1). For this, reads from these samples were downloaded, trimmed, and sub-sampled to the lowest number of reads, as according to Weinheimer and Aylward [56]. CoverM was used with the same parameters and coverage cutoffs described above and the same RPKM calculation (Supplementary Dataset 3).

### Visualizations, statistical analyses, and sequence benchmarking

All plots aside from the maps of Fig. 1 were created in R (version 3.5.1) [57] with RStudio (version 1.1.456) [58] using vegan [59], ggpubr [60], and ggplot2 (3.1.1) [61]. Maps were created with QGIS (3.24) using the Voyager plug-in for the base and overlaid with sample data. Because statistics and trends held regardless of protein examined per bacteria or phage (Supplementary Dataset 4), we focused on the TerL results to represent phage diversity and COG85 results to represent bacterial diversity, as these genes were the most prevalent in the dataset (Supplemental Dataset 2). Influential sequences and physicochemical parameters were identified by those varying the most with variation in the communities of all samples based on significant vector length (vegan package function envfit, perm=999, na.rm=TRUE; calculated with |NMDS1-NMDS2|; *p* values < 0.01). Distance decay analyses were performed by calculating the pairwise, geodesic distance of samples using their longitude and latitude (package: geodist) and the community composition distances of samples were based on Bray–Curtis (vegdist(distance = ”bray”)). The spatial distance was correlated with compositional distance between samples with Pearson correlations (cor.test(method = ”pearson”,alternative = ”two.sided”)). A regression was plotted with geom_smooth(method= “lm”) from ggplot2 in R. The correlation statistics are reported in Supplementary Dataset 4 under the tab “DistanceDecay”. Distance decay was visualized with ggplot2 by plotting the spatial distance in kilometers against the Bray–Curtis distance of samples in Fig. 2c, e. Community composition of samples were compared and visualized in non-metric dimensional scaling (NMDS) plots using Bray–Curtis distances of relative abundances calculated with reads per kilobase per million (RPKM) using vegan (metaMDS (distance = “bray”)). Two outlier samples were excluded in the community compositional analyses as these were highly divergent (WAM_TWN phage community and EPM_13A1 phage and prokaryote communities) which skewed the ordination (Supplementary Figs. 4, 6a). WAM_TWN was sampled in a highly polluted site, and EPM_13A1 was sampled from a completely freshwater sample, which likely resulted in their aberrant community compositions at the genus-level (Supplementary Fig. 5). Ellipses in NMDS plot Fig. 4 was drawn with the ggplot2 function stat_ellipse(type = “norm”), and significant environmental variables were calculated with envfit(permutations = 999) of the Bray–Curtis distance matrix of a sample and the environmental variables of the sample. The output (envift_output) was converted to a dataframe with (as.data.frame(scores(envfit_output,”vectors”)) * ordiArrowMul(envfit_output) and plotted onto the ggplot2 with geom_segment with the linetype corresponding to the significance (*p* values 0.01–0.05: dashed, *p* values < 0.01: solid). Significant distinctions between oceans and habitat types were determined with PERMANOVA tests (vegan package) based on Bray–Curtis dissimilarity matrices using the RPKM data (adonis2 function). Mantel tests were performed with the Bray–Curtis distance matrices between samples using each sequence’s RPKM tables calculated with vegdist(method = ”bray”) and running the function mantel(method= “pearson”, permutations=9999). Shannon’s Diversity index and Simpson’s Diversity index were calculated with the distance() function from the vegan package, and sample results are reported in Supplementary Dataset 1. Shannon’s Diversity is reported in this study, but the Simpson’s index yielded the same significance of statistical tests reported in the main text.

### Gene taxonomy

Prokaryotic sequences corresponding to COG85 were classified via centrifuge [46]. For the phages, amino acid sequences of TerL genes were aligned to RefSeq 207 [29] with LAST [62] (lastal -m 10 -f BlastTab; E value cutoff 10^−5^), and the taxonomy of the hit’s host was reported (i.e. a hit to a *Prochlorococcus* phage meant the taxonomy of *Prochlorococcus* was reported) Supplementary Dataset 5. The top hit was detected based on percent identity. The top 10 genera based on average relative abundance across samples was reported.

### Supplementary information

Supplementary Information

Supplementary Dataset 1

Supplementary Dataset 2

Supplementary Dataset 3

Supplementary Dataset 4

Supplementary Dataset 5

## Data Availability

Reads from metagenomes were deposited on the European Nucleotide Archive with the accession codes ERS17080717 - ERS17080773 under the project accession PRJEB70438. Sequences of marker genes and phage contigs can be found on the GitHub repository Panama-Phage-and-Prokaryotes-Diversity (https://github.com/scubalaina/Panama-Prokaryotes-and-Phage-Diversity/tree/main), along with the VOG and COG HMM profiles used for marker gene detection. Other data from the manuscript are located in the Supplementary Materials.

## Code availability

Custom scripts used for this study are found in the GitHub repository (https://github.com/scubalaina/Panama-Prokaryotes-and-Phage-Diversity/tree/main).
