# Supplementary material for: Contrasting drivers of abundant phage and prokaryotic communities revealed in diverse coastal ecosystems
Source: ISME Commun. 2023 Dec 4;3:127. doi: 10.1038/s43705-023-00333-6 (PMC10695958; doi:10.1038/s43705-023-00333-6)
Supplement: Supplementary file 1 — Supplementary Information [file 43705_2023_333_MOESM1_ESM.pdf]

Supplementary Information for Weinheimer et al. Contrasting drivers of abundant phage and prokaryotic communities revealed in diverse coastal ecosystems

Supplementary Figure 1-6 captions

Supplementary Figure 1. Maps of phage or prokaryotic sequences of the reefs of this study found at a Tara station in the sample of the SRF layer (3 - 7m) and 0.22-3um size fraction.

Supplementary Figure 2. Boxplot of the number of Tara Ocean stations that a phage or prokaryotic sequence of the reefs from this study is found.

Supplementary Figure 3. Correlogram with the rows as the environmental factor and the columns as the Shannon's Diversity of the phages or prokaryotes by each environment of this study.

Supplementary Figure 4. NMDS plots based on Bray Curtis Similarities between samples in the EP mangrove including sample 13A1.

Supplementary Figure 5. Composition of the dominant genera for the putative hosts of phages and prokaryotes in each sample.

Supplementary Figure 6. NMDS plots of the WA communities including outlier samples WAM_TWN.

Supplementary Dataset captions

Supplementary Dataset 1. Dataset with information on sample collection, environmental measurements, metagenome statistics, and Tara Oceans sample metadata from [1].

Supplementary Dataset 2. Dataset with the table of VOGs from VOG version 208 used for the MCP and TerL detection; output tables from phage contig detection tools VirSorter2 and CheckV; summary table of contig detection results

Supplementary Dataset 3. Tables of the RPKM of each sequence type (TerL, MCP, phage contig, COG85, COG86, COG12) in the Panama metagenomes of this study and the Tara Oceans metagenomes.

Supplementary Dataset 4. Tables of ecological statistical test outputs (e.g. Mantel test *p values*, Shannon's Diversity correlations) of each sequence type examined (TerL, MCP, phage contig, COG85, COG86, COG12).

Supplementary Dataset 5. Tables of TerL and COG85 sequence classification, distribution statistics in the Panama metagenomes of this study (e.g. number of mangrove samples); tables of TerL and COG85 distribution in the Tara Oceans metagenomes.

Supplementary Discussion

- *Genus composition*

Supplementary Figures 1 - 6

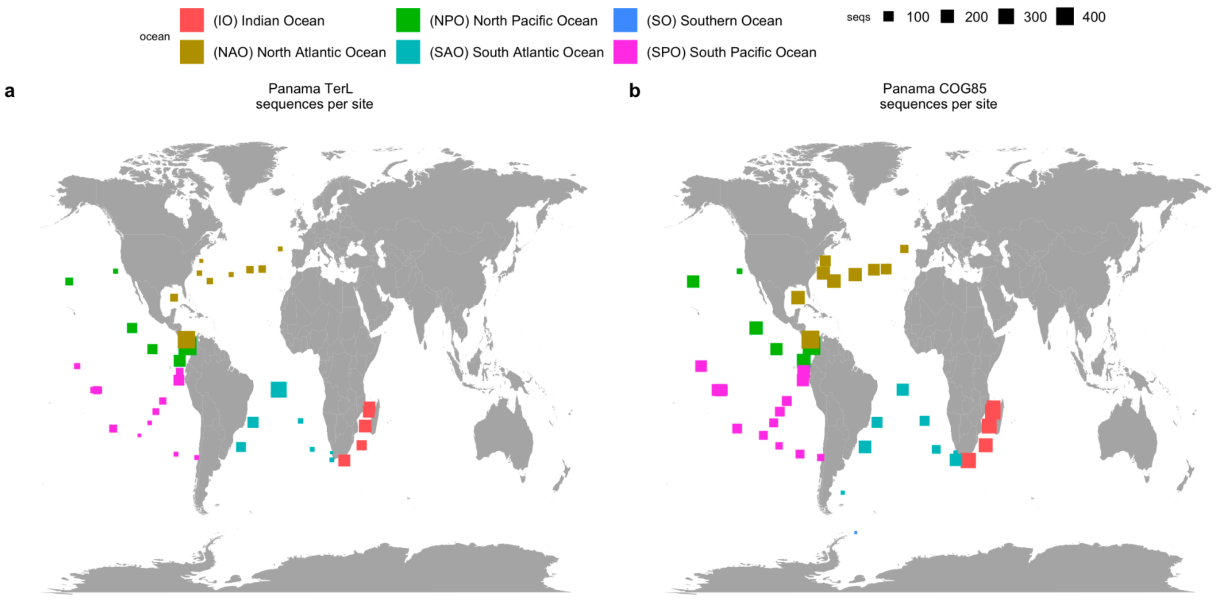

Supplementary Figure 1. Maps of phage or prokaryotic sequences of the reefs of this study found at a Tara station in the sample of the SRF layer (3 – 7 m) and 0.22-3um size fraction. (**a**) Number of TerL sequences and (**b**) COG85 sequences found at each Tara station examined. Made with data from Supplementary Dataset 3. Point colors correspond to oceanic region, and point size corresponds to the number of sequences at a station.

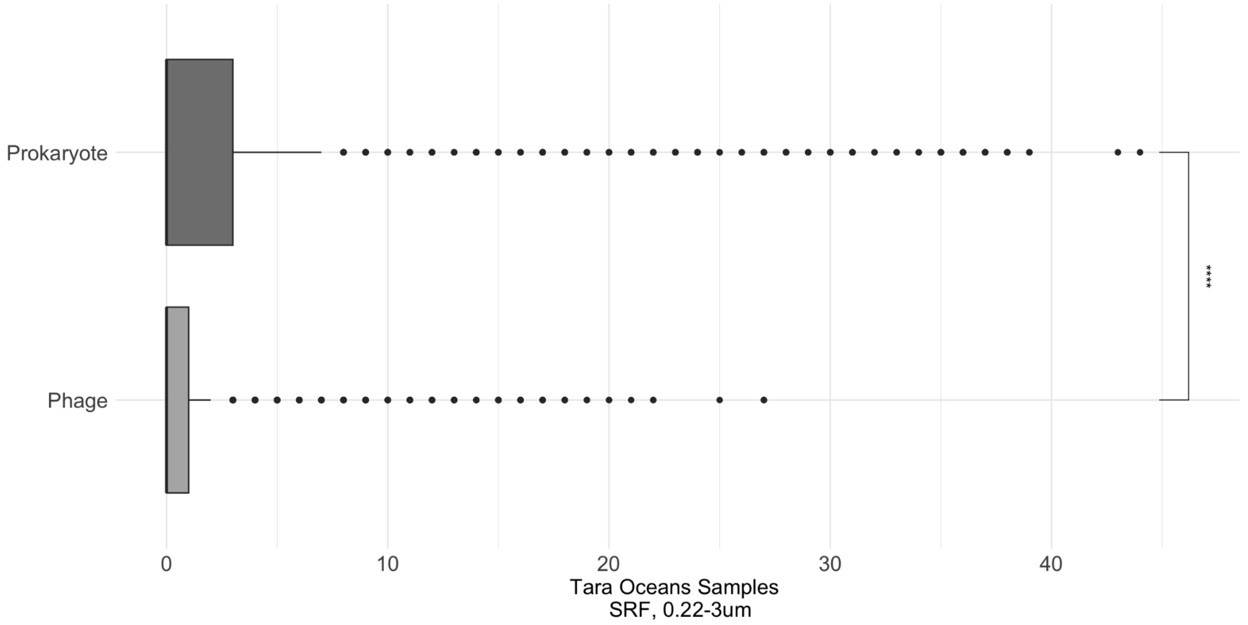

Supplementary Figure 2. Boxplot of the number of Tara Ocean stations that a phage or prokaryotic sequence of the reefs from this study is found. **** corresponds to Wilcox test *p value* < 0.0001.

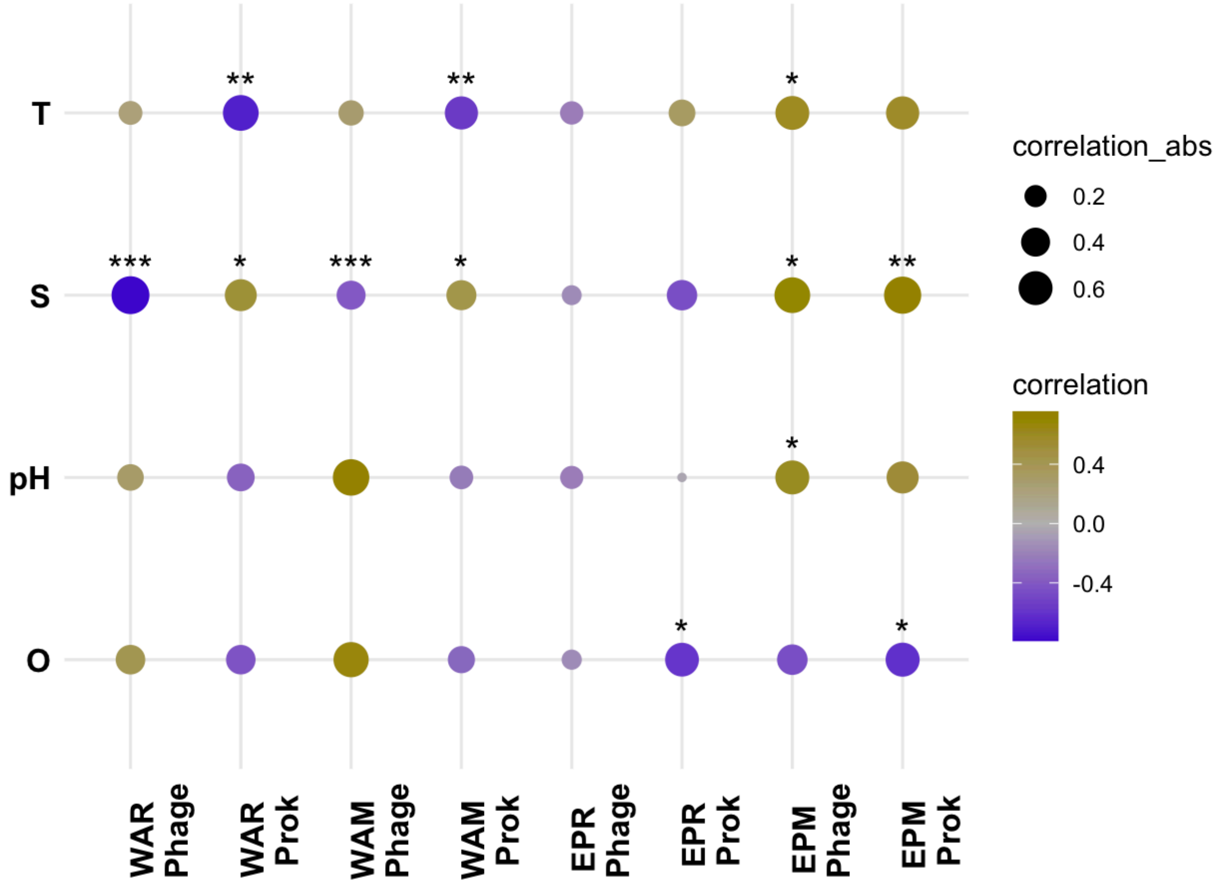

Supplementary Figure 3. Correlogram with the rows as the environmental factor and the columns as the Shannon's Diversity of the phages (Phage) or prokaryotes (Prok) by each environment of this study (WAR, WAM, EPR, EPM). Strength of correlation indicated by size and color. Direction of correlation indicated by color (purple - negative, gold - positive). Stars correspond to Pearson correlation p values (* < 0.05, ** < 0.01, *** < 0.001). T- temperature, S - salinity, O - dissolved oxygen.

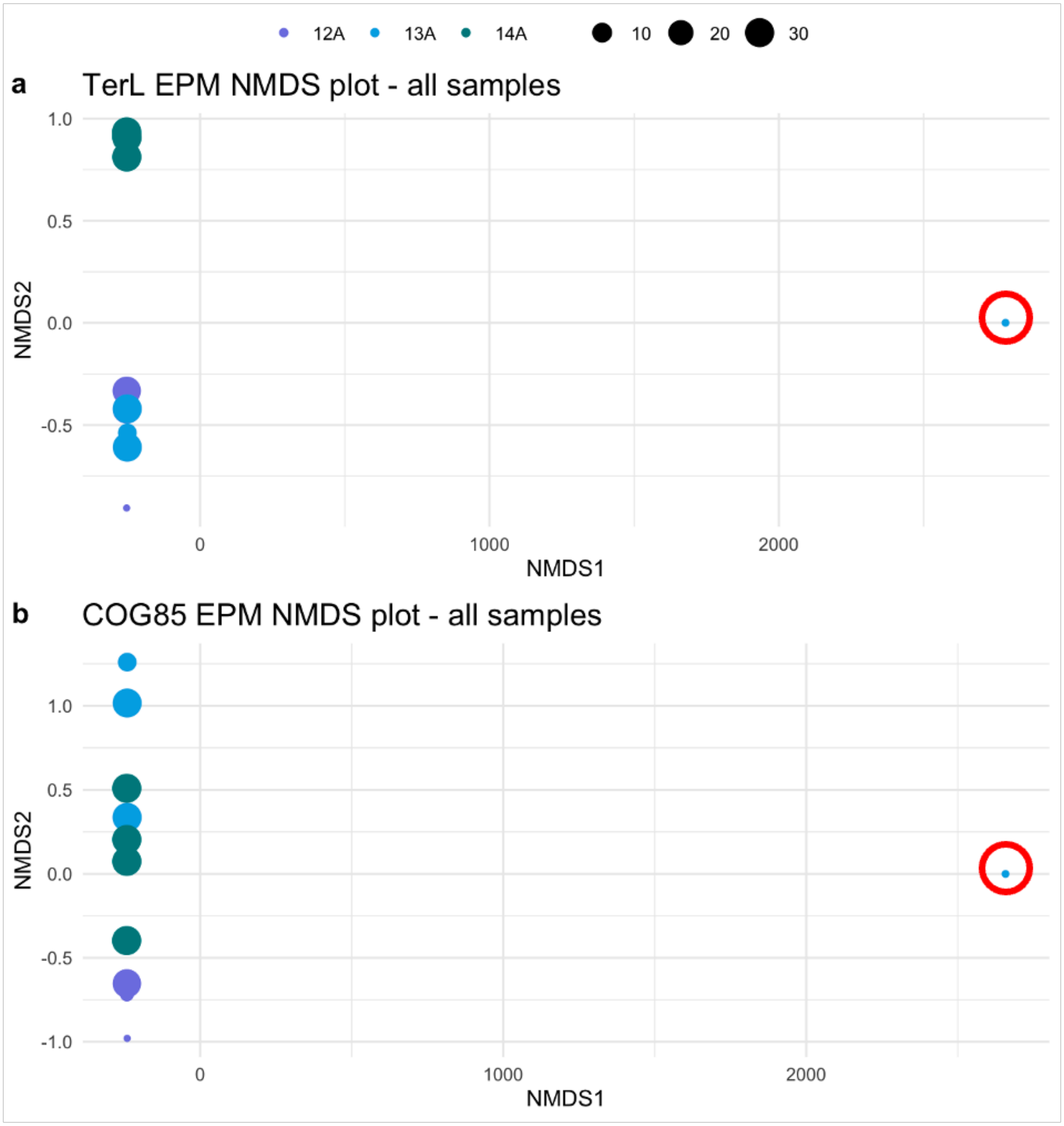

Supplementary Figure 4. NMDS plots based on Bray Curtis Similarities between samples in the EP mangrove including sample 13A1. (a) NMDS plot of phage community composition. (b) NMDS plot of prokaryotic community composition. Color of point corresponds to river. Size of point corresponds to salinity. Red circles around outlier sample EPM_13A1.

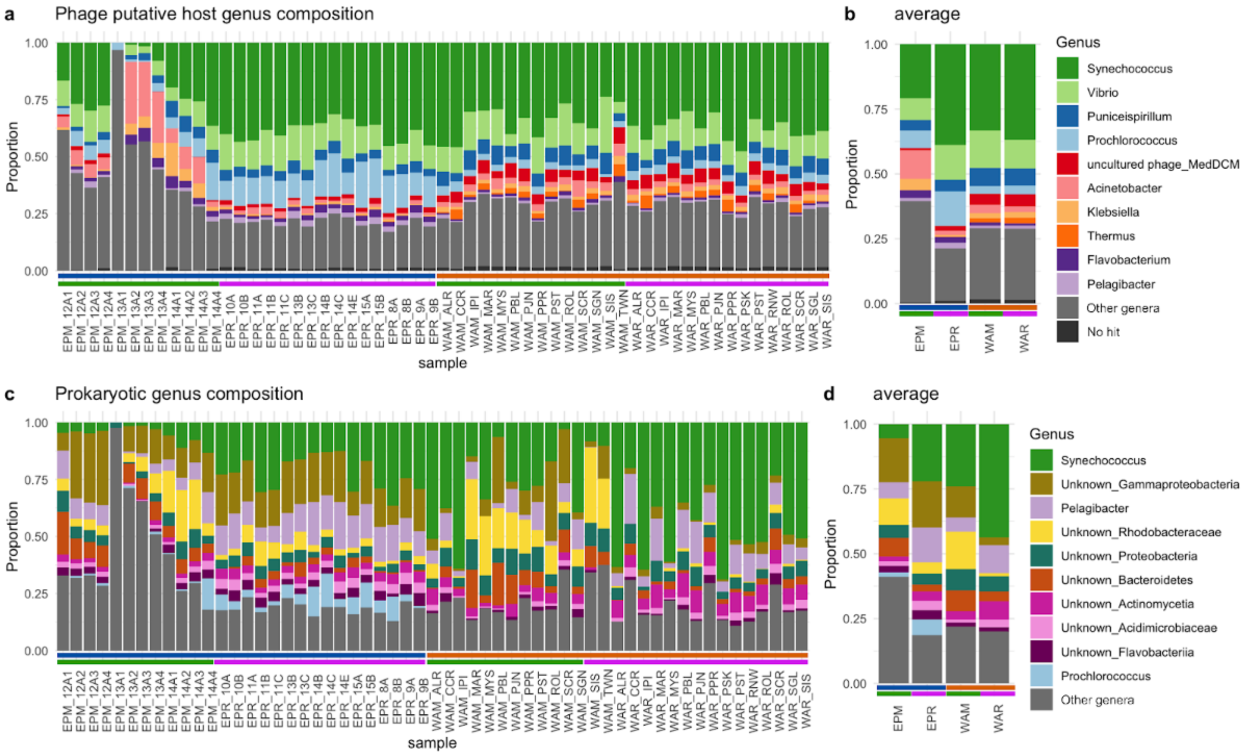

Supplementary Figure 5. Composition of the dominant genera for the putative hosts of phages and prokaryotes in each sample. (**a,c**) Composition of the top then most common genera of the putative host of the phages (**a**) and the prokaryotes (**c**) in each sample. (**b,d**) Average composition of the putative hosts of phages (**b**) or of the prokaryotes (**d**) in each environment. Top color strip corresponds to ocean (EPR - blue, WAR orange), bottom corresponds to habitat type (mangrove - green, reef - magenta).

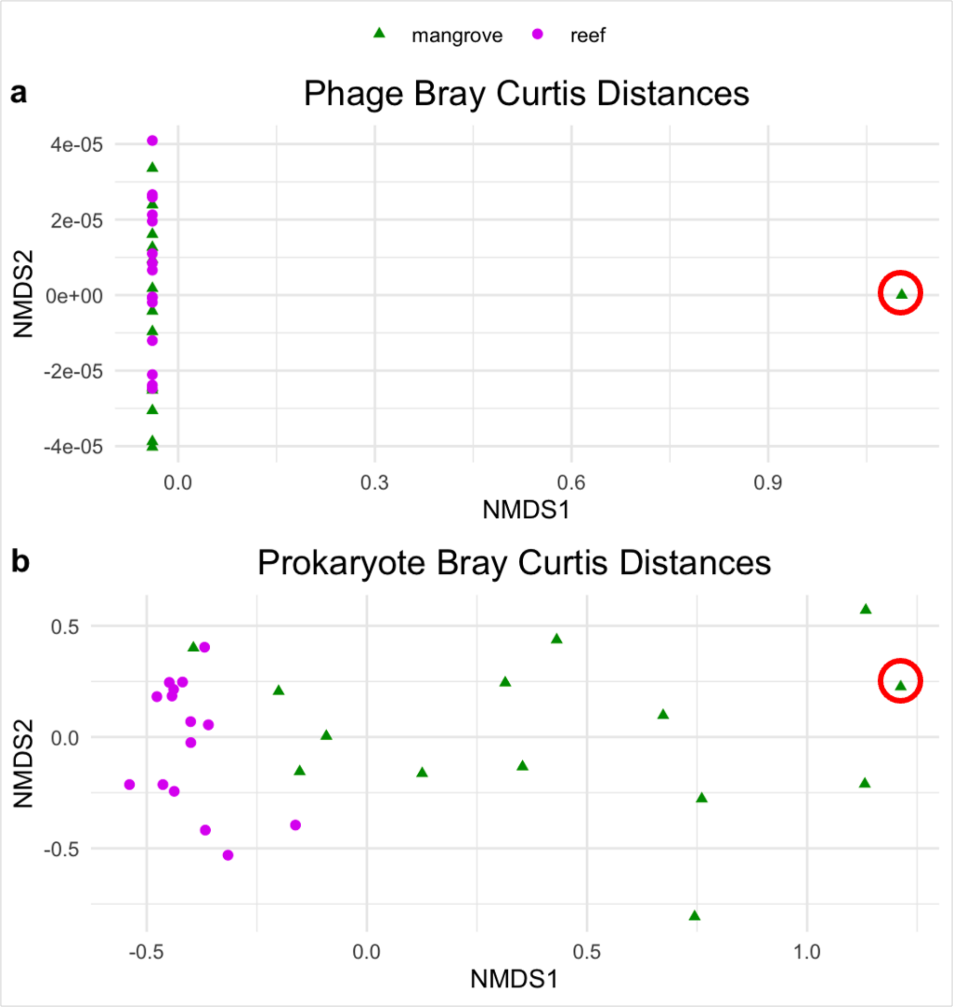

Supplementary Figure 6. NMDS plots of the WA communities including outlier samples WAM_TWN. (a) Phage community composition ordination using the TerL gene. (b) Prokaryotic community composition using the COG85 gene. Point color and shape correspond to habitat type. Red circle is around the outlier sample WAM_TWN.

Supplementary Discussion

Genus composition

Within the WA, the average genus composition of both the putative phage hosts and of the prokaryotes corroborate the compositional distinctions observed above when using sequence-level composition (Figure 4a,c). The putative host genera of the phage communities were highly similar between WAM and WAR (Supplementary Figure 5), and the genera composition of the prokaryotes being quite distinct between the WAM and WAR (Supplementary Figure 5) with an average enrichment in genera belonging to genera of the Rhodobacteraceae family, Bacteroidetes phylum, and Gammaproteobacteria class within the WAM, and an average enrichment of prokaryotes belonging to the *Synechococcus* genus, *Pelgaibacter* genus, and Actinomycetia class in the WAR.

Within the EPR and EPM, both putative phage host taxa and prokaryotic taxa were enriched *Prochlorococcus* relative to the WAR and WAM, which highlights the more pelagic waters of the EP as *Prochlorococcus* is known to be more dominant in pelagic waters compared to the than coastal waters of the WA, where *Synechococcus* is prevalent [2]. Notably, the fully freshwater sample EPM_13A only contained *Prochlorococcus* of the top genera in the putative host community for the phages (Supplementary Figure 5). *Prochlorococcus* bacteria are rarely found in brackish or freshwater conditions [3, 4], and instead, a *Prochlorococcus*-like bacteria that is larger in cell size than its marine counterpart has been reported in estuaries [4]. Thus, the presence of this phage terminase with homology to that of a *Prochlorococcus* phage in the fully fresh sample suggests that either (i) this phage infects this *Prochlorococcus*-like freshwater bacteria, (ii) that it has a broad host range that enables it to infect marine and freshwater bacteria, (iii) or that its homology is a result of the limitation of the reference database. Interestingly, the other fully freshwater sample, EPM_12A1, contained a community that was a bit more similar to the more saline EPM samples, potentially due to more mixing, but flow rates were not measured and thus remains uncertain. The prokaryotic community in EPM_13A1 freshwater sample contained only an unknown genus in the Proteobacteria phylum that was also prevalent in the other samples (Supplementary Figure 5c), which is unsurprising as diverse Proteobacteria are common in freshwater systems [5], and similarly to the putative hosts of the phages, the other freshwater sample of the EPM_12A1 had taxa more similar to the marine samples, but notably much less Gammaproteobacteria than the other EPM samples in that river (EPM12A2-4). The notable divergence of the genera in this freshwater sample for both the prokaryotes and putative host community of the phages highlights the crucial role of salinity in shaping microbial communities [6, 7].

Supplementary Discussion references

1. Sunagawa S, Coelho LP, Chaffron S, Kultima JR, Labadie K, Salazar G, et al. Ocean plankton. Structure and function of the global ocean microbiome. Science 2015; 348: 1261359.
2. Differential distribution and ecology of *Prochlorococcus* and *Synechococcus* in oceanic waters.
3. Vaulot D, Partensky F, Neveux J, Mantoura RFC, Llewellyn CA. Winter presence of prochlorophytes in surface waters of the northwestern Mediterranean Sea. Limnol Oceanogr 1990; 35: 1156–1164.
4. Shang X, Zhang LH, Zhang J. Prochlorococcus-like populations detected by flow cytometry in the fresh and brackish waters of the Changjiang Estuary. J Mar Biol Assoc U K 2007; 87: 643–648.
5. Zwart G, Crump BC, Kamst-van Agterveld MP, Hagen F, Han SK. Typical freshwater bacteria: an analysis of available 16S rRNA gene sequences from plankton of lakes and rivers. Aquat Microb Ecol 2002; 28: 141–155.
6. Logares R, Bråte J, Bertilsson S, Clasen JL, Shalchian-Tabrizi K, Rengefors K. Infrequent marine-freshwater transitions in the microbial world. Trends Microbiol 2009; 17: 414–422.
7. Cabello-Yeves PJ, Rodriguez-Valera F. Marine-freshwater prokaryotic transitions require extensive changes in the predicted proteome. Microbiome 2019; 7: 117.
